# Analysis of the Dick Effect for AI-Based Dynamic Gravimeters

**DOI:** 10.3390/s25237167

**Published:** 2025-11-24

**Authors:** Wen-Zhang Wang, Xi Chen, Jin-Ting Li, Dan-Fang Zhang, Wei-Hao Xu, Jia-Yi Wei, Jia-Qi Zhong, Biao Tang, Lin Zhou, Jin Wang, Ming-Sheng Zhan

**Affiliations:** 1 Innovation Academy for Precision Measurement Science and Technology, Chinese Academy of Sciences, Wuhan 430071, China; wangwz@apm.ac.cn (W.-Z.W.); lijinting@apm.ac.cn (J.-T.L.); zhangdf@apm.ac.cn (D.-F.Z.); xuweihao@apm.ac.cn (W.-H.X.); weijy@apm.ac.cn (J.-Y.W.); jqzhong@apm.ac.cn (J.-Q.Z.); biaotang@apm.ac.cn (B.T.); lzhou@apm.ac.cn (L.Z.); mszhan@apm.ac.cn (M.-S.Z.); 2 School of Physical Sciences, University of Chinese Academy of Sciences, Beijing 100049, China; 3 Wuhan Institute of Quantum Technology, Wuhan 430206, China; 4 Hefei National Laboratory, Hefei 230088, China

**Keywords:** atom interferometer, accelerometer, dynamic gravity measurement, gravimeter, gravity noise analysis, Dick effect

## Abstract

Atom interferometer (AI)-based dynamic gravimeters enable high-precision absolute gravity measurements, which are crucial for applications in geophysics, navigation, resource exploration, and metrology. Understanding their underlying mechanisms and minimizing measurement noise is essential for enhancing performance. This work investigates gravity-measurement noise in AI-based systems induced by the dead time of the classical accelerometer. Using actual dynamic gravity-measurement data, we demonstrate that a dead time of 0.12 s introduces significant gravity-measurement noise, reaching 8 mGal. To elucidate the mechanism of this noise, we derive a frequency-domain formula, identifying high-frequency aliasing as its source. Analysis of the derived expressions indicates that reducing the dead-time duration and suppressing the acceleration’s high-frequency noise are effective strategies for mitigating this noise. This work provides significant insights into noise analysis and the future design of AI-based dynamic gravimeters.

## 1. Introduction

Dynamic gravity measurement is an important method for obtaining gravity fields with high precision and high spatial resolution, with significant applications in geophysics [1], navigation [2], resource exploration [3,4], and metrology [5,6]. A dynamic atomic gravimeter is a hybrid gravimeter that combines the measurements of an atom interferometer and a classical accelerometer, and can achieve absolute gravity measurement with high precision. The dynamic atomic gravity measurement method originates from the vibration compensation scheme used in static atomic gravity measurements [7], which reduces environmental vibration noise. This method was later extended to dynamic environments [8,9,10,11,12,13,14]. In 2011, Geiger et al. demonstrated dynamic atomic acceleration measurement in a 0 g plane [15]. In 2013, Bidel et al. realized atomic gravity measurement in an elevator [16]. In 2018 and 2020, Bidel et al. demonstrated dynamic atomic absolute gravity measurements on a ship and an airplane, and achieved superior gravity-measurement precision compared to the traditional spring dynamic gravimeter [17,18,19]. In 2022, Guo et al. used a dynamic atomic gravimeter on moving vehicles [20,21]. In 2023 and 2025, Wu et al. demonstrated dynamic gravity measurement on ships and then in aircraft [22,23,24,25,26]. Chen et al. also developed a dynamic atomic gravimeter and carried out dynamic gravity measurement on a lake in 2021 and on the sea in 2024 [27,28]. The developed gravimeter showed precision at the sub-mGal level.

Improving precision and elucidating the measurement mechanisms are hot topics in AI-based dynamic gravimeters. Xu et al. proposed a method to optimize the transfer function of the accelerometer [29]. Zhou et al. proposed a method to suppress the cross-coupling effect [28]. Cheiney et al. proposed a Kalman-filter algorithm to reduce measurement noise [30], which has been applied by several research groups for dynamic gravity data processing [31,32]. Huang et al. modeled the temperature of an accelerometer to suppress its drift [33].

An AI-based dynamic gravimeter combines an AI and a classical accelerometer. The AI measures acceleration using a free-falling cold atom cloud. This measured acceleration is absolute; however, the data are discrete, and its measurement range is limited. The classical accelerometer is solidly connected to the AI and measures acceleration synchronously. This measured acceleration is continuous, and its measurement range is large; however, it has unknown offset and drift. The combination of both sensors enables continuous, absolute gravity acceleration measurements with a wide range [34].

The Dick effect is a well-known phenomenon in atomic frequency standards, first comprehensively analyzed for passively operated atomic clocks [35,36,37,38,39,40]. Its core mechanism is the aliasing of high-frequency noise due to the periodic and discontinuous nature of the measurement. In a typical atomic clock, the local oscillator (LO) is continuously running, but its frequency is only periodically corrected based on the error signal derived from probing an atomic resonance at discrete times. The key issue arises from the dead time between successive atomic interrogations. During this dead time, the LO’s phase noise evolves uncorrected. The frequency stability of the clock, characterized by its Allan deviation, is degraded because the high-frequency components of the LO’s phase noise are not measured directly. Instead, through the process of periodic sampling, this high-frequency noise is aliased down into the frequency band near DC.

While the Dick effect was originally formalized for atomic clocks, its fundamental principle—aliasing induced by periodic, discontinuous measurement—is universal and directly applicable to other quantum sensors, including AI-based gravimeters. A critical link is the sampling process. Dynamic gravity measurement extracts weak gravity signals from a strong acceleration background. The magnitude of gravity variation is on the order of mGal (10^−5^ m/s^2^), while the magnitude of the acceleration background is usually on the order of m/s^2^. Suppression of the acceleration noise by five orders of magnitude is achieved by using a low-pass filter (LPF). Unlike the classical Dick effect in atomic clocks, this effect in atomic interferometers involves both “sampling” and “filtering” processes, which is also where the Dick effect in dynamic atomic gravimeters differs from that of atomic clocks.

In atomic interferometry gravimeter systems, although continuous acceleration acquisition can be achieved through an FPGA, certain data acquisition systems (e.g., NI-based architectures) may result in incomplete acceleration sampling when triggered by the interferometer sequence. This issue arises from uncertainty in the interference-cycle period and the time required for data storage, thereby introducing noise into dynamic gravity measurements. Addressing this phenomenon is one of the key motivations of our work. Furthermore, our study alerts practitioners to the potential influence of discontinuous acceleration sampling on the accuracy of dynamic gravity measurements—a question that has remained unexplored in previous literature. This work provides a detailed analysis of the resulting effects.

This article is organized as follows. In Section 2, the principle and the data-processing procedures of AI-based dynamic gravity measurement are introduced. By introducing the dead time of the acceleration, the gravity-measurement noise is calculated in the time domain using actual dynamic gravity-measurement data. In Section 3, by analyzing the frequency spectrum of acceleration, the formula of the dead-time-induced gravity noise is derived, the physical mechanism for this noise is explained, the dead-time-induced gravity noise is calculated using actual dynamic gravity-measurement data, and the noise distribution over frequency is illustrated. In Section 4, the conclusions and a discussion are given.

## 2. Time-Domain Analysis of Dead-Time-Induced Gravity-Measurement Noise

### 2.1. Principle of Dynamic Hybrid Measurement and How Dead Time Is Induced

**Figure 1:**
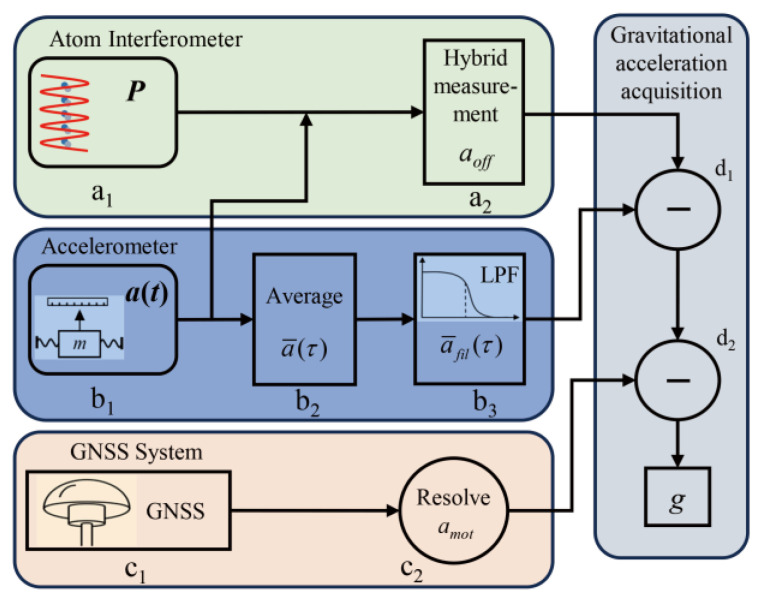
Schematic of the AI-based dynamic gravity-measurement principle. a_1_–a_2_: atomic interferometer process; b_1_–b_3_: accelerometer procedure; c_1_–c_2_: GNSS operation flow; d_1_–d_2_: gravity-signal acquisition.

The dynamic hybrid gravity-measurement principle is illustrated in Figure 1. In the AI, a cold atom cloud is subjected to π/2–π–π/2 Raman laser pulses, forming an interference loop. The population *P* is measured by fluorescence detection. The classical accelerometer simultaneously measures the acceleration of the AI platform but with an unknown offset. This acceleration, defined as *a*_cla_, is used in conjunction with a sensitivity function [41] over the interference time interval to calculate the compensation phase *φ*_com_. The interference fringe is then reconstructed by plotting *φ*_com_ against the population *P*. Sine fitting is used to fit this fringe, and the obtained phase is defined as the offset phase *φ*_off_. By using *φ*_off_, the acceleration offset of the classical accelerometer *a*_off_ is obtained. The absolute acceleration *a*_abs_ experienced by the AI is derived as *a*_abs_ = *a*_cla_ − *a*_off_.

**Figure 2:**
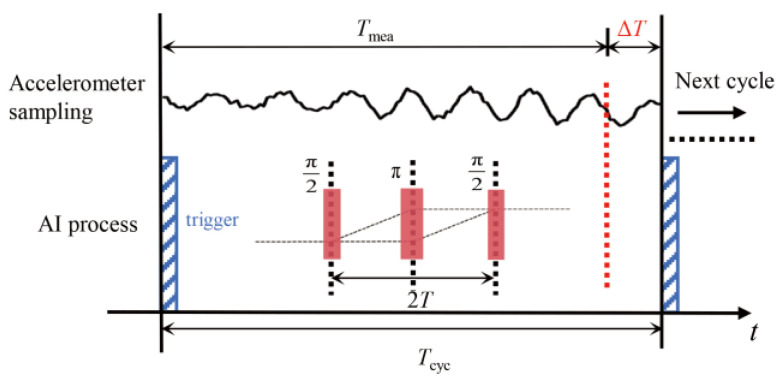
Acquisition timeline of the classical acceleration *a*_cla_(*t*), showing the dead time Δ*T* within one AI experiment cycle. The horizontal axis represents the synchronous measurement sequence of the accelerometer and the AI. The upper curve represents the acceleration measured by the accelerometer, and the lower curve represents the AI process.

The hybrid dynamic gravity-measurement process requires a continuous classical acceleration sequence, typically obtained by averaging *a*_cla_(*t*) over AI measurement cycles. For synchronization in hybrid gravity measurement, the acquisition of the classical acceleration is triggered by the AI’s time sequence. Due to trigger preparation and data-processing overhead, the measurement time *T*_mea_ of *a*_cla_(*t*) within one experimental cycle does not fully cover the AI cycle time *T*_cyc_, as illustrated in Figure 2. The missing sampling time Δ*T* = *T*_cyc_ − *T*_mea_ is defined as the dead time. For convenience in the subsequent theoretical analysis and calculations, the dead time Δ*T* is assumed to occur at the end of the cycle. This paper investigates the gravity-measurement noise induced by Δ*T*.

A typical example is the NI-based acquisition system we previously used, which uses the interferometer timing to trigger the accelerometer. Due to the uncertainty of the interference timing period and the time requirements for storing acceleration data, the acceleration data is incomplete. Within a cycle of 600 ms, there is a dead time of 100 ms. Consequently, we adopted an FPGA-based acquisition scheme, which can achieve non-dead-time acceleration acquisition throughout the entire interference cycle. In our dynamic gravity-measurement process, acceleration data without dead time was collected, but in order to study the dead-time effect, some data points were manually removed.

### 2.2. Dynamic Gravity-Measurement Data-Processing Flow

The corrected acceleration is used directly for dynamic gravity output following the procedure in Figure 1. To analyze the impact of dead time, the basic data-processing flow for gravity measurement is introduced.

The corrected acceleration *a*_cla_ has a sampling rate of 50 kHz. To investigate the dead-time effect, the raw acceleration data were stored. To minimize storage requirements, the data were averaged and downsampled to 250 Hz. For the acceleration signal *a*(*t*) at an interference period of *T*_cyc_ = 0.6 s (corresponding to 150 sampling points), selected data points were removed to simulate the effect of dead time. Subsequently, the data was averaged within each individual interference cycle. This averaging step aimed to maintain temporal continuity in the presence of dead time, rather than filter out noise in specific frequency bands. This resulted in a processed acceleration signal $\bar{a}(\tau )$ with a sampling frequency of 1/*T*_cyc_ ≈ 1.67 Hz.

In marine dynamic gravity measurement, the typical period of ship-motion acceleration is approximately 10 s. Since the averaged signal has a much shorter period, it cannot suppress noise at this 10-s period. To mitigate this noise, a 300-s low-pass filter was applied to $\bar{a}(\tau )$. The filtered acceleration, denoted as ${\bar{a}}_{fil}(\tau )$, included contributions from gravity and the Coriolis acceleration due to the Earth’s rotation. This motion-induced component is denoted *a*_mot_. To remove *a*_mot_, the ship’s latitude and longitude were recorded in real time using GNSS, and the motion acceleration was calculated and subtracted according to the method described in Ref. [18]. The final gravity value *g* is given by *g* = ${\bar{a}}_{fil}(\tau )$ − *a*_mot_. Given a ship speed of 10 knots and a filter time constant of 300 s, the corresponding spatial resolution of the gravity measurement is approximately 1.5 km. Gravity variations at higher frequencies cannot be resolved with this method.

### 2.3. Source of Dynamic Gravity Data

**Figure 3:**
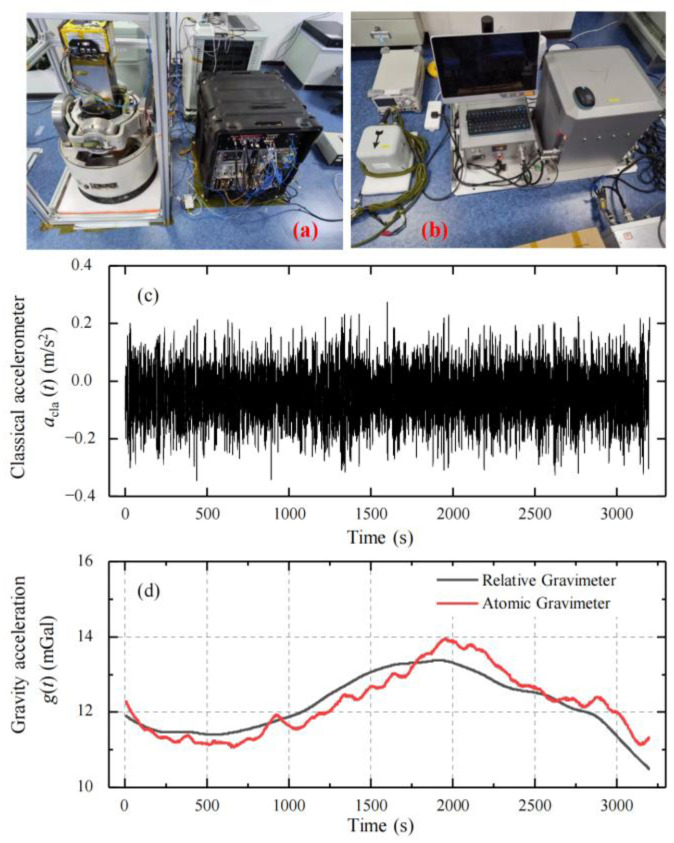
AI-based dynamic gravimeter setup and measurement data. (**a**) The AI-based dynamic gravimeter. (**b**) The spring dynamic gravimeter. (**c**) Acceleration *a*_cla_ collected by the classical accelerometer. (**d**) The measured gravity values from both gravimeters. Both measured gravity values were subtracted using the same normal gravity model for convenience of display.

To study the dead-time effect, we analyzed the measurement data from our self-developed dynamic gravimeter, as shown in Figure 3a. For comparison, a spring dynamic gravimeter was installed on the same ship (as shown in Figure 3b), which measured the gravity synchronously. An acceleration acquisition method was developed to avoid dead time, enabling the collection of all acceleration data in an AI measurement cycle. Marine dynamic gravity measurements followed the procedure in Figure 1, using the full-sampled acceleration to extract the gravity signal. The gravity survey was along a line at a speed of about 10 knots, and the length of the line was about 45 km. The measurement results can be found in Ref. [29], and a subset is shown in Figure 3. The acceleration *a*_cla_ recorded by the classical accelerometer is shown in Figure 3c, with a peak-to-valley value of about 0.6 m/s^2^. The measured gravity values are also shown in Figure 3d. The measured gravity value is shown in Figure 3d. The standard deviation of the difference between the gravity measurements from the two gravimeters was 0.4 mGal. The AI-based dynamic gravimeter’s results in Figure 3d, being highly precise, were used as a gravity reference *g*_ref_(*t*) for studying the dead-time-induced gravity-measurement noise.

### 2.4. Method and Results of Dynamic Gravity-Measurement Data with Dead Time

**Figure 4:**
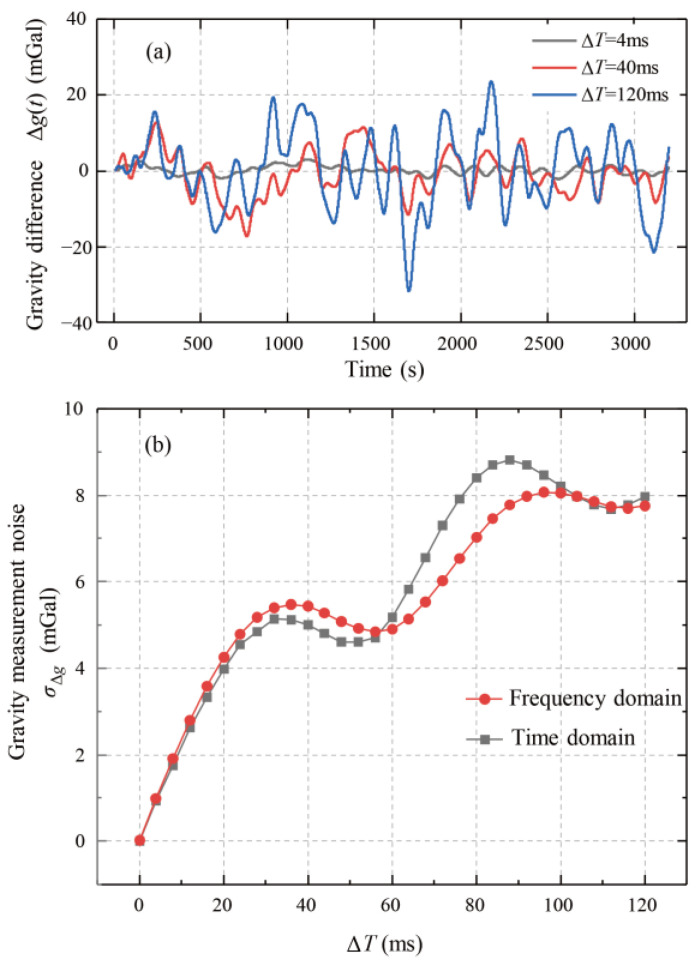
Gravity-measurement noise induced by dead time Δ*T* with the AI cycle time *T*_cyc_ = 0.6 s. (**a**) Gravity difference Δ*g*(*t*) between *g*_und_(*t*) and *g*_ref_(*t*). (**b**) Gravity-measurement noise σ*_Δg_* over Δ*T* calculated in the time domain (black square) and increasing acceleration measurement noise over Δ*T* calculated in the frequency domain (red circle).

The gravity-measurement noise with dead time was quantified by removing the data points at the end of each cycle. In Figure 1, procedures b_1_-b_3_ differ between the cases with and without dead time. The procedure shown in Figure 1 was then followed to calculate the undersampled gravity value *g*_und_(*t*). The gravity value *g*_und_(*t*) was compared with the reference *g*_ref_(*t*) to obtain the gravity difference Δ*g*(*t*) = *g*_und_(*t*) − *g*_ref_(*t*). The calculated Δ*g*(*t*) for different Δ*T* is shown in Figure 4a. The gravity difference fluctuated significantly as Δ*T* increased. The standard deviation of Δ*g*(*t*), denoted by σ_Δ*g*_, represents the gravity-measurement noise. The relationship between σ_Δ*g*_ and Δ*T* is shown in Figure 4b. As the dead time Δ*T* increased, the gravity difference fluctuation σ_Δ*g*_ also increased. The noise σ_Δ*g*_ reached 1 mGal for a short dead time of Δ*T* = 4 ms and increased to 8 mGal for Δ*T* = 100 ms. This calculation demonstrates that dead time causes non-negligible gravity-measurement noise, leading to severe degradation of measurement precision.

## 3. Frequency-Domain Analysis of Dead-Time-Induced Gravity-Measurement Noise

While the preceding time-domain analysis can quantify the gravity noise, it cannot physically explain the mechanism and magnitude of the noise generation. For a steady-state random signal, the time-domain signal is random, but its power-spectrum density (PSD) has a stable profile. Starting from the PSD of the acceleration signal, this section derives the analytical relationship between the gravity noise and the acceleration PSD to better analyze the mechanism of this effect.

**Figure 5:**
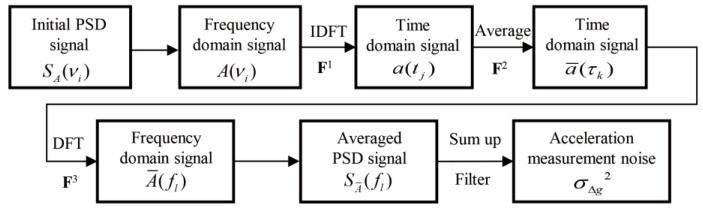
Procedures for deriving the relationship between the acceleration PSD $S_{A}(\nu_{i})$ and the gravity-measurement noise ${\sigma_{∆g}}^{2}$.

The derivation procedure is outlined in Figure 5. Starting from the initial acceleration PSD, the corresponding frequency-domain signal is transformed to the time domain to analyze the averaging process. The averaged time-domain signal is then transformed back to the frequency domain to analyze the low-pass filter process. From this frequency-domain signal, the PSD of the acceleration after averaging and filtering is calculated. The gravity noise is subsequently calculated based on this PSD. We note that the derived relationship depends on the initial PSD but is independent of the specific characteristics of the random time-domain acceleration signal. The detailed calculation procedure is shown in the figure below.

### 3.1. Derivation Between Gravity Noise and PSD

The initial PSD of the acceleration is denoted as $S_{A}(\nu_{i})$, (*i* = 1…*n*). The PSD corresponds to a time-domain signal with *n* data points and a sampling interval of *T*_sam_. Consequently, the PSD also has *n* data points. The frequency interval Δ*ν* is 1/(*nT*_sam_), and the range of *ν_i_* is (−1/2*T*_sam_, 1/2*T*_sam_). Due to the symmetry |*A*(*ν_i_*)| = |*A*(−*ν_i_*)|, subsequent calculations consider only the non-negative frequencies (0, 1/2*T*_sam_). The relationship between $S_{A}(\nu_{i})$ and the corresponding discrete frequency-domain signal $A(\nu_{i})$ is given by

(1) $$A\left(\nu_{i}\right)={\left[n\cdot S_{A}\left(\nu_{i}\right)\right]}^{1/2}$$

The discrete time-domain signal $a(t_{j})$ (*j* = 1…*n*) is obtained by the inverse discrete Fourier transform (IDFT). The time interval of *t_j_* is *T*_sam_,

(2) $$a(t_{j})=\frac{1}{\sqrt{n}}\sum_{i=1}^{n}A(\nu_{i})\cdot e^{-2\pi I(i-1)(j-1)/n}$$

The measured acceleration *a*(*t_j_*) is averaged over each cycle time *T*_cyc_, and the averaged acceleration is denoted by $\bar{a}(\tau_{k})$ (*k* = 1…*m*), where *k* is the index of counting the measurement cycles of $\bar{a}$, $\tau_{k}$ is the timestamp of the *k*-th averaging cycle, *m* = *n*/*c* is the number of averaged acceleration points, and *c* = *T*_cyc_/*T*_sam_ is the number of data points per cycle. Due to the dead time, $a(t_{j})$ is only averaged over the measurement time *T*_mea_, and *c*_mea_ = *T*_mea_/*T*_sam_ is the number of effective data points averaged per cycle. The averaging is expressed as

(3) $$\bar{a}(\tau_{k})=\frac{1}{c_{mea}}\sum_{j=(k-1)c+1}^{(k-1)c+c_{mea}}a(t_{j})$$

The signal $\bar{a}(\tau_{k})$ is then transformed into the frequency domain, yielding $\bar{A}(f_{l})$ (*l* = 1…*m*), where $f_{l}$ denotes the frequency of the averaged acceleration frequency-domain signal, distinguishing it from the frequency *ν_i_* of the initial acceleration frequency-domain signal:

(4) $$\bar{A}(f_{l})=\frac{1}{\sqrt{m}}\sum_{k=1}^{m}\bar{a}(\tau_{k})\cdot e^{2\pi I(k-1)(l-1)/m}$$

The interval of *f_l_* is Δ*f* = 1/*nT*_sam_, and the range of *f_l_* is (0, 1/2*T*_cyc_). Similarly, the relationship between $\bar{A}(f_{l})$ and its PSD is given by $S_{\bar{A}}(f_{l})=\frac{1}{m}\cdot {\left|\bar{A}(f_{l})\right|}^{2}$. Substituting Equations (2)–(4) into the PSD definition yields

(5) $$S_{\bar{A}}(f_{l})=c\sum_{i=1}^{n}H_{l,i}S_{A}(\nu_{i})$$

where $H_{l,i}$ represents the transfer function between the PSDs. The detailed derivation of $H_{l,i}$ is illustrated in Appendix A and Appendix B.

For computational convenience in the filtering process, an ideal (rectangular) low-pass filter is used. This is implemented by setting $S_{\bar{A}}(f_{l})$ to zero for all $f_{l}$ above the cut-off frequency. Thus, the filtered acceleration measurement error variance ${\sigma_{\bar{a}\_fil}}^{2}$ is

(6) $${\sigma_{\bar{a}\_fil}}^{2}=\sum_{l=1}^{l_{fil}}S_{\bar{A}}(f_{l})$$

where *l*_fil_ = ⌊*nT*_sam_/*T*_fil_⌋ is the frequency index corresponding to the low-pass filter’s cut-off frequency 1/*T*_fil_, and the symbol ⌊ ⌋ represents the floor function. By substituting Equation (6) into Equation (5), we have

(7) $${\sigma_{\bar{a}\_fil}}^{2}=c\sum_{l=1}^{l_{fil}}\sum_{i=1}^{n}H_{l,i}\;S_{A}(\nu_{i})$$

The variance ${\sigma_{\bar{a}\_fil}}^{2}$ includes the desired low-frequency gravity signal and the motion acceleration (<1/*T*_fil_ Hz). However, we wish to calculate the gravity noise originating from the aliasing of the high-frequency acceleration components (>1/*T*_fil_ Hz) to the low-frequency range (<1/*T*_fil_ Hz) through $H_{l,i}$. Therefore, the gravity-measurement noise ${\sigma_{∆g}}^{2}$ is obtained by excluding the intrinsic low-frequency signal component:

(8) $${\sigma_{∆g}}^{2}=c\sum_{l=1}^{l_{\mathrm{fil}}}\sum_{i=m+1}^{n}H_{l,i}\;S_{A}(\nu_{i})$$

Equation (8) represents the fundamental relationship between the gravity-measurement noise and the acceleration signal’s PSD that we set out to derive.

### 3.2. Analysis of the Mechanism of Dead-Time-Induced Gravity Noise

Appendix B provides the expression of the matrix element $H_{l,i}$ of **H**. The matrix **H** has the characteristics described below.

First, the matrix **H** is constructed by the horizontal concatenation of *c* diagonal matrices ${\tilde{H}}^{p}$, where *p* = 0, 1 …, *c*−1 indexes the sub-matrices, and each matrix ${\tilde{H}}^{p}$ has dimensions *m* × *m*. This structure is detailed in Equation (9), with the first line showing the layout, and the second line specifying the values of the diagonal elements:

(9) $$\left\{\begin{array}{l} H=\left\{{\tilde{H}}^{0}{\tilde{H}}^{1}\cdots {\tilde{H}}^{p}\cdots {\tilde{H}}^{c}\right\}\\ {\tilde{H}}_{l,l}^{p}=H_{l,pm+l} \end{array}\right.$$

In Equation (5), $S_{A}(\nu_{i})$ has a length of *n* and a frequency range of (0, 1/2*T*_sam_), while $S_{\bar{A}}(f_{l})$ has a length of *m* and a frequency range of (0, 1/2*T*_cyc_). Thus, the sub-matrix ${\tilde{H}}^{p}$ maps the PSD elements $S_{A}(\nu_{i})$ from the frequency range (*p*/*T*_cyc_, (*p* + 1)/*T*_cyc_) to the PSD $S_{\bar{A}}(f_{l})$ in the baseband (0, 1/2*T*_cyc_).

After filtering, as shown in Equation (7), the variation of ${\sigma_{\bar{a}\_fil}}^{2}$ accumulates contributions from $S_{\bar{A}}(f_{l})$ only for *l* ≤ *l*_fil_. Therefore, for a given *p*, the elements *H_l,i_*(*i* = *pm* + *l*) that contribute to ${\sigma_{\bar{a}\_\mathrm{fil}}}^{2}$ are those where *pm* < *i* < *pm* + *l*_fil_. This implies that only PSD components $S_{A}(\nu_{i})$ within the frequency range (*p*/*T*_cyc_, *p*/*T*_cyc_ + *l*_fil_/*nT*_sam_) contribute to ${\sigma_{\bar{a}\_fil}}^{2}$.

**Figure 6:**
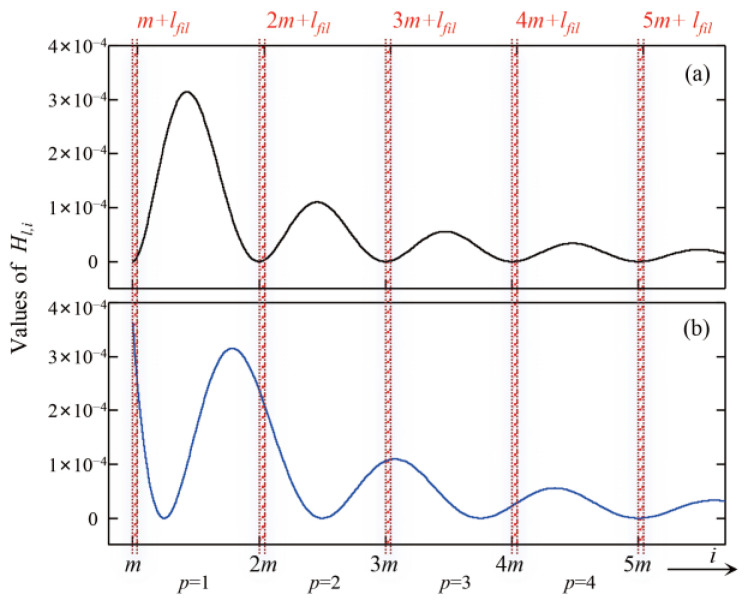
Values of *H_l,i_* (*i* = *pm* + *l*) for *p* > 1. The red-shaded area indicates the range *pm* < *i* < *pm* + *l*_fil_, corresponding to the low-pass filter’s passband. (**a**) Δ*T* = 0, (**b**) Δ*T* = 0.12 s.

Second, the value of *H_l,__i_* (*i* = *pm* + *l*) for *p* > 0 is shown in Figure 6. When Δ*T* = 0, the values of *H_l,i_* for *pm* < *i* <*pm* + *l*_fil_ are nearly zero. In contrast, when Δ*T* ≠ 0, these values deviate from zero, thereby inducing gravity noise ${\sigma_{∆g}}^{2}$, as given by Equation (8).

Thus, the mechanism is clear: high-frequency aliasing, induced by incomplete acceleration sampling, is the source of the increased gravity-measurement noise.

### 3.3. Verification with Actual PSD and Noise Distribution

**Figure 7:**
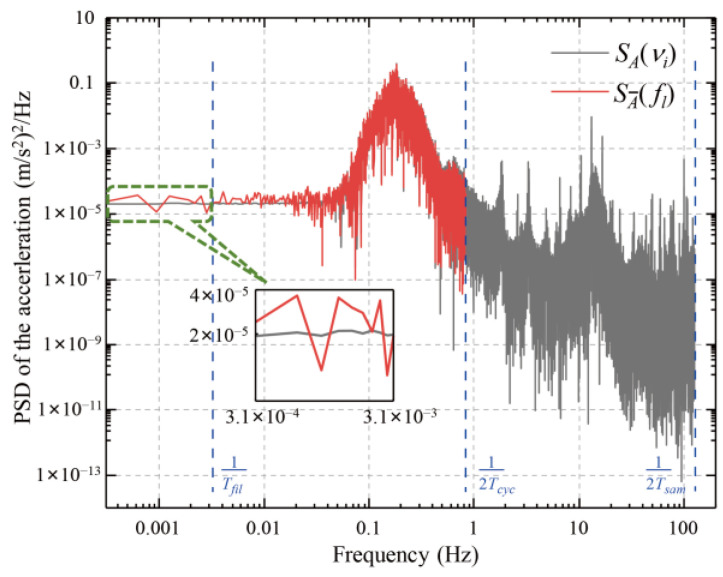
Calculated PSDs: the initial acceleration $S_{A}(\nu_{i})$ and the averaged acceleration $S_{\bar{A}}(f_{l})$ for Δ*T* = 0.12 s.

To verify the correctness of the derived equation, the actual acceleration data *a*(*t_j_*) from Figure 3c was used to calculate its PSD $S_{A}(\nu_{i})$. The PSD $S_{\bar{A}}(f_{l})$ of the averaged acceleration $\bar{a}(\tau_{k})$ for Δ*T* = 0.12 s was then calculated using Equation (5), as shown in Figure 7. The Nyquist frequencies for these two PSDs were 125 Hz and 0.83 Hz, respectively. The two PSDs exhibit similar shapes in the frequency range of 0.05 Hz to 0.83 Hz. This is because the dominated components of $S_{A}(\nu_{i})$ are concentrated in this band, being about two orders of magnitude higher than the components in other frequency bands. Consequently, the aliasing effect due to Δ*T* does not significantly alter the shape in this band. However, in the lower-frequency range (DC–0.05 Hz), the PSD of $S_{\bar{A}}(f_{l})$ is significantly higher and noisier than $S_{A}(\nu_{i})$. This elevated noise floor is the aliasing effect caused by Δ*T*. It is confirmed that when Δ*T* = 0, $S_{\bar{A}}(f_{l})$ and $S_{A}(\nu_{i})$ are identical in this low-frequency band.

The gravity-measurement noise $\sigma_{∆g}$ was then calculated using Equation (8) for different Δ*T*, where the filtering time of 300 s corresponded to *l*_fil_ = 10. The results are shown in Figure 4b. As anticipated from the frequency-domain approach, the calculated noise amplitude matches the $\sigma_{∆g}$ obtained in the time-domain analysis. The slight discrepancies are likely due to the different filter types used: a Bessel filter was applied for time-domain processing, whereas an ideal rectangular filter was assumed in the frequency-domain derivation for simplicity. Nevertheless, the overall trend and amplitude of $\sigma_{∆g}$ agree well, verifying the correctness of the derivation equations in Section 3.1. Figure 4b shows that if dead time is unavoidable, reducing its duration is an effective way to mitigate the induced gravity-measurement noise.

The contributions of different frequency bands can be analyzed. The contributions of the sub-matrices ${\tilde{H}}^{p}$ to $\sigma_{∆g}$ were calculated to analyze the noise components in different frequency bands. The components of $\sigma_{∆g}$ induced by the *p*-th frequency band via ${\tilde{H}}^{p}$ were calculated as

(10) $${\sigma_{∆g\_p}}^{2}=c\sum_{l=1}^{l_{\mathrm{fil}}}\sum_{i=pm+1}^{(p+1)m}H_{l,i}\;S_{A}(\nu_{i})$$

**Figure 8:**
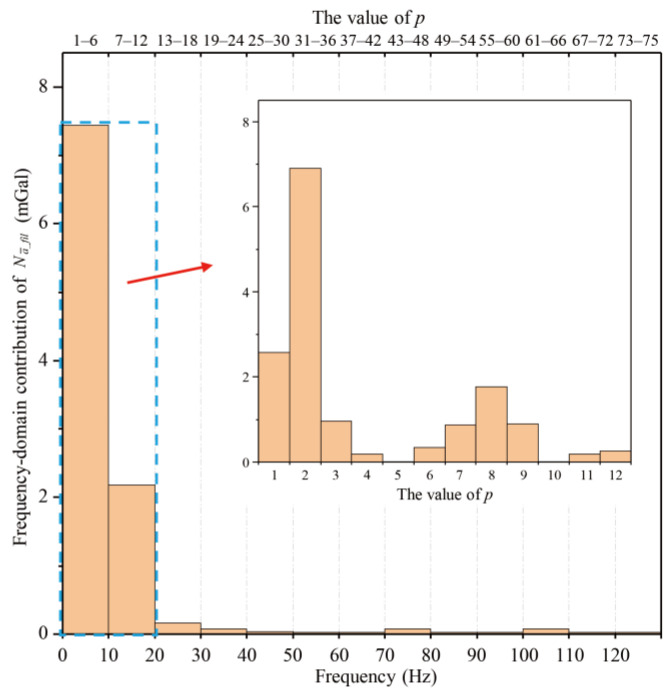
Contribution of different frequency bands to the gravity-measurement noise $\sigma_{∆g}$ for Δ*T* = 0.12 s. The inset shows an enlarged view of the contributions in the 0~20 Hz band.

For ${\tilde{H}}^{p}$, the corresponding frequency range of the input PSD is (*p*/*T*_cyc_, (*p*+1)/*T*_cyc_). For our parameters, this is approximately (*p* × 1.7 Hz, *p* × 1.7 + *l*_fil_ × Δ*ν* Hz). Figure 8 shows $\sigma_{∆g\_p}$ for Δ*T* = 0.12 s. The highest contribution comes from *p* = 2, with a value of about 7 mGal originating from the frequency band approximately between 3.33 Hz and 5 Hz. Prominent peaks are observed at around 1.7 Hz and 3.3 Hz in the PSD $S_{A}(\nu_{i})$ in Figure 7. Therefore, suppressing high-frequency acceleration noise is another effective strategy to reduce $\sigma_{∆g}$ if Δ*T* cannot be eliminated.

## 4. Conclusions and Discussion

This paper investigates the AI-based dynamic gravity-measurement noise induced by the Dick effect. The role of dead time in the classical acceleration measurement is introduced. We found that dead time indeed causes gravity-measurement noise, which was first quantified in the time domain. The results show that even a short dead time of 4 ms (0.67% of the 0.6 s cycle) can cause 1 mGal of gravity-measurement noise. It is therefore essential for practitioners to ensure continuous acquisition to avoid such noise. To clarify the underlying mechanism and derive an analytical expression for this noise, formulas relating the PSD of the averaged acceleration and the resulting noise to the initial acceleration PSD were derived. Analysis using the derived formulas confirmed that high-frequency aliasing is the mechanism underlying the dead-time-induced gravity noise. The PSD of the averaged acceleration and the resulting gravity noise were calculated using actual acceleration data. The close agreement between the time-domain and frequency-domain noise calculations verifies the correctness of the derived formulas. Our analysis reveals that a dead time of 0.12 s can induce a significant gravity-measurement noise of 8 mGal. Meanwhile, reducing the dead-time duration and suppressing the high-frequency noise of the classical accelerometer are two effective methods to mitigate this gravity noise.

This work elucidates the mechanism of Dick effect-induced noise in AI-based dynamic gravity measurements and provides practical suppression methods. It holds significant importance for both noise analysis and the design schemes of AI-based dynamic gravimeters operating on dynamic platforms. Furthermore, the derived formulation is generally applicable for noise analysis involving averaging, filtering, and undersampling processes.

## Data Availability

The data that support the findings of this study are available from the corresponding author upon reasonable request.

## Appendix A

To calculate the conversion relationship between $S_{A}(\nu_{i})$ and $S_{\bar{A}}(f_{l})$, first calculate the relationship between the frequency-domain signals $A(\nu_{i})$ and $\bar{A}(f_{l})$, and define $\bar{A}\left(f_{l}\right)=D_{l,i}A(\nu_{i})$, where $H_{l,i}={\left|D_{l,i}\right|}^{2}$. The operations of IDFT, averaging, and DFT in Equations (2)–(4) are represented as the matrices **F**^1^, **F**^2^, and **F**^3^, and substituting these matrices into Equation (4) leads to

(A1) $$\bar{A}(f_{l})=\sum_{i=1}^{n}D_{l,i}A(\nu_{i})$$

where **D** = **F**^3^**F**^2^**F**^1^ is the frequency-transfer matrix and $D_{l,i}$ is the element in the matrix **D**. Directly calculating the matrix **D** = **F**^3^**F**^2^**F**^1^ is rather complicated. A stepwise approach is adopted. First, calculate the matrix **F**^4^ = **F**^2^**F**^1^, where the matrix element of **F**^1^ is

(A2) $$F_{j,i}^{1}=\frac{1}{\sqrt{n}}e^{\beta (i-1)(j-1)}$$

“I” denotes the imaginary unit (I^2^ = −1), and **F**^2^ represents the averaging process. Matrix multiplication yields

(A3) $$F^{4}=\frac{1}{c_{mea}}\frac{1}{\sqrt{n}}\left(\begin{array}{ccc} F_{1,1}^{4} & \ldots & F_{1,n}^{4}\\ \vdots & F_{k,i}^{4} & \vdots \\ F_{m,1}^{4} & \ldots & F_{m,n}^{4} \end{array}\right)$$

and then calculating **D** = **F**^3^**F**^4^ gives

(A4) $$D=F^{3}F^{4}=\frac{1}{\sqrt{m}}\frac{1}{c_{mea}}\frac{1}{\sqrt{n}}\left(\begin{array}{ccc} F_{1,1}^{3} & \ldots & F_{1,m}^{3}\\ \vdots & F_{l,k}^{3} & \vdots \\ F_{m,1}^{3} & \ldots & F_{m,m}^{3} \end{array}\right)\left(\begin{array}{ccc} F_{1,1}^{4} & \ldots & F_{1,n}^{4}\\ \vdots & F_{k,i}^{4} & \vdots \\ F_{m,1}^{4} & \ldots & F_{m,n}^{4} \end{array}\right)$$

Similarly, performing matrix multiplication gives

(A5) $$D=\left(\begin{array}{ccc} D_{1,1} & \ldots & D_{1,n}\\ \vdots & D_{l,i} & \vdots \\ D_{m,1} & \ldots & D_{m,n} \end{array}\right)$$

where the elements of the matrix **D** are

(A6) $$D_{l,i}=\frac{1}{n}\frac{\sqrt{c}}{c_{mea}}\frac{1-e^{\beta c_{mea}(i-1)}}{1-e^{\beta (i-1)}}\frac{1-e^{\beta n(i-l)}}{1-e^{\beta c(i-l)}};l\in [1,m],i\in [1,n]$$

Equation (A6) contains the factor $\frac{1-e^{\beta n(i-l)}}{1-e^{\beta c(i-l)}}$, which expands to $\frac{1-e^{-2\pi I(i-l)}}{1-e^{-2\pi I(i-l)c/n}}$. When *i* = *pm* + *l*,

(A7) $$\frac{1-e^{-2\pi I(i-l)}}{1-e^{-2\pi I(i-l)c/n}}=\frac{1-e^{-2\pi I*pm}}{1-e^{-2\pi I*p}}$$

Equation (A7) becomes indeterminate at specific frequencies i = pm + l. However, a limit of this expression at these points exists. Now, add a tiny quantity δ to calculate its limit value. After expansion and calculation,

(A8) $$\underset{\delta \to 0}{lim}\frac{1-e^{-2\pi I*p[m+\delta ]}}{1-e^{-2\pi I*p[1+\delta ]}}=m$$

Therefore, when *i* = *pm* + *l*, *D_l,i_* has a non-zero value, whereas when *i* ≠ *pm* + *l*, the denominator $1-e^{\beta c(i-l)}$ is non-zero, while the numerator $1-e^{\beta n(i-l)}$ is zero, resulting in *D_l,i_* being zero.

## Appendix B

As established in Appendix A, the matrix **D** is characterized by sub-matrices with non-zero diagonal elements. Denote $H={\left|D\right|}^{2}$, and the values of the **H** matrix elements are given by the following formula:

(A9) $$\sum_{i=1}^{n}H_{l,i}=\frac{1}{n}\cdot \sum_{i=1}^{n}{\left|D_{l,i}\right|}^{2}$$

It follows that the matrix **H** inherits the same structural form as the matrix **D**, meaning its sub-matrices also possess non-zero diagonal elements. The specific values of the elements $H_{l,i}={\left|D_{l,i}\right|}^{2}$ are determined as follows:

(A10) $$\left\{\begin{array}{ll} H_{l,i}=\frac{1}{c}\frac{1}{{c_{mea}}^{2}}\frac{1-\cos[2\pi c_{mea}\frac{i-1}{n}]}{1-\cos[2\pi \frac{i-1}{n}]}; & i=pm+l,\\ H_{l,i}=0; & i\neq pm+l. \end{array}\right.$$

## References

[1] Velicogna I., Wahr J. (2006). Measurements of Time-Variable Gravity Show Mass Loss in Antarctica. Science.

[2] Battelier B., Barrett B., Fouché L., Chichet L., Antoni-Micollier L., Porte H., Napolitano F., Lautier J., Landragin A., Bouyer P. Development of Compact Cold-Atom Sensors for Inertial Navigation. Proceedings of the SPIE Photonics Europe.

[3] Nabighian M.N., Ander M.E., Grauch V.J.S., Hansen R.O., LaFehr T.R., Li Y., Pearson W.C., Peirce J.W., Phillips J.D., Ruder M.E. (2005). Historical Development of the Gravity Method in Exploration. Geophysics.

[4] Davis K., Li Y., Batzle M. (2008). Time-Lapse Gravity Monitoring: A Systematic 4D Approach with Application to Aquifer Storage and Recovery. Geophysics.

[5] Cladé P., De Mirandes E., Cadoret M., Guellati-Khélifa S., Schwob C., Nez F., Julien L., Biraben F. (2006). Determination of the Fine Structure Constant Based on Bloch Oscillations of Ultracold Atoms in a Vertical Optical Lattice. Phys. Rev. Lett..

[6] Lamporesi G., Bertoldi A., Cacciapuoti L., Prevedelli M., Tino G.M. (2008). Determination of the Newtonian Gravitational Constant Using Atom Interferometry. Phys. Rev. Lett..

[7] Le Gouët J., Mehlstäubler T.E., Kim J., Merlet S., Clairon A., Landragin A., Pereira Dos Santos F. (2008). Limits to the Sensitivity of a Low Noise Compact Atomic Gravimeter. Appl. Phys. B.

[8] Merlet S., Le Gouët J., Bodart Q., Clairon A., Landragin A., Pereira Dos Santos F., Rouchon P. (2009). Operating an Atom Interferometer beyond Its Linear Range. Metrologia.

[9] Barrett B., Antoni-Micollier L., Chichet L., Battelier B., Lévèque T., Landragin A., Bouyer P. (2016). Dual Matter-Wave Inertial Sensors in Weightlessness. Nat. Commun..

[10] Ménoret V., Vermeulen P., Le Moigne N., Bonvalot S., Bouyer P., Landragin A., Desruelle B. (2018). Gravity Measurements below 10^−9^ g with a Transportable Absolute Quantum Gravimeter. Sci. Rep..

[11] Gong W., Li A., Huang C., Che H., Feng C., Qin F. (2022). Effects and Prospects of the Vibration Isolation Methods for an Atomic Interference Gravimeter. Sensors.

[12] Che H., Li A., Zhou Z., Gong W., Ma J., Qin F. (2023). An Approach of Vibration Compensation for Atomic Gravimeter under Complex Vibration Environment. Sensors.

[13] Gong W., Li A., Luo J., Che H., Ma J., Qin F. (2023). A Vibration Compensation Approach for Atom Gravimeter Based on Improved Sparrow Search Algorithm. IEEE Sens. J..

[14] Qiao Z., Shen Z., Hu R., Li L., Yuan P., Wu G., Yuan Y., Zhou Y., Wu B., Lin Q. (2025). A Vibration Compensation Approach for Shipborne Atomic Gravimeter Based on Particle Swarm Organization. Sci. Rep..

[15] Geiger R., Ménoret V., Stern G., Zahzam N., Cheinet P., Battelier B., Villing A., Moron F., Lours M., Bidel Y. (2011). Detecting Inertial Effects with Airborne Matter-Wave Interferometry. Nat. Commun..

[16] Bidel Y., Carraz O., Charrière R., Cadoret M., Zahzam N., Bresson A. (2013). Compact Cold Atom Gravimeter for Field Applications. Appl. Phys. Lett..

[17] Bidel Y., Zahzam N., Blanchard C., Bonnin A., Cadoret M., Bresson A., Rouxel D., Lequentrec-Lalancette M.F. (2018). Absolute Marine Gravimetry with Matter-Wave Interferometry. Nat. Commun..

[18] Bidel Y., Zahzam N., Bresson A., Blanchard C., Cadoret M., Olesen A.V., Forsberg R. (2020). Absolute Airborne Gravimetry with a Cold Atom Sensor. J. Geod..

[19] Bidel Y., Zahzam N., Bresson A., Blanchard C., Bonnin A., Bernard J., Cadoret M., Jensen T.E., Forsberg R., Salaun C. (2023). Airborne Absolute Gravimetry With a Quantum Sensor, Comparison With Classical Technologies. JGR Solid Earth.

[20] Guo J., Ma S., Zhou C., Liu J., Wang B., Pan D., Mao H. (2022). Vibration Compensation for a Vehicle-Mounted Atom Gravimeter. IEEE Sens. J..

[21] Zhang J.-Y., Xu W.-J., Sun S.-D., Shu Y.-B., Luo Q., Cheng Y., Hu Z.-K., Zhou M.-K. (2021). A Car-Based Portable Atom Gravimeter and Its Application in Field Gravity Survey. AIP Adv..

[22] Zhou Y., Zhang C., Chen P., Cheng B., Zhu D., Wang K., Wang X., Wu B., Qiao Z., Lin Q. (2023). A Testing Method for Shipborne Atomic Gravimeter Based on the Modulated Coriolis Effect. Sensors.

[23] Wu B., Zhang C., Wang K., Cheng B., Zhu D., Li R., Wang X., Lin Q., Qiao Z., Zhou Y. (2023). Marine Absolute Gravity Field Surveys Based on Cold Atomic Gravimeter. IEEE Sens. J..

[24] Qiao Z.-K., Yuan P., Zhang J.-J., Zhang Z.-Y., Li L.-L., Zhu D., Jiang M.-R., Shi H.-Y., Hu R., Zhou F. (2023). Error Analysis and Filtering Methods for Absolute Ocean Gravity Data. IEEE Sens. J..

[25] Chen P., Peng S., Wang J., Gao M., Lv X., Yang D., Jin Z., Wang Y., Tang X., Wu B. (2025). An Airborne Design of Inertial Stabilized Platform for Cold Atom Gravimeter. IEEE Sens. J..

[26] Zhai C., Wang J., Zhou J., Wang Y., Tang X., Zhou Y., Zhang C., Li R., Shu Q., Wang K. (2025). Airborne absolute gravity measurements based on quantum gravimeter. Acta Phys. Sin..

[27] Ge G., Chen X., Li J., Zhang D., He M., Wang W., Zhou Y., Zhong J., Tang B., Fang J. (2023). Accuracy Improvement of a Compact 85Rb Atom Gravimeter by Suppressing Laser Crosstalk and Light Shift. Sensors.

[28] Zhou Y., Wang W., Ge G., Li J., Zhang D., He M., Tang B., Zhong J., Zhou L., Li R. (2024). High-Precision Atom Interferometer-Based Dynamic Gravimeter Measurement by Eliminating the Cross-Coupling Effect. Sensors.

[29] Xu A., Kong D., Fu Z., Wang Z., Lin Q. (2019). Vibration Compensation of an Atom Gravimeter. Chin. Opt. Lett..

[30] Cheiney P., Fouché L., Templier S., Napolitano F., Battelier B., Bouyer P., Barrett B. (2018). Navigation-Compatible Hybrid Quantum Accelerometer Using a Kalman Filter. Phys. Rev. Appl..

[31] Zhu D., Xu H., Zhou Y., Wu B., Cheng B., Wang K.-N., Chen P.-J., Gao S.-T., Weng K.-X., Wang H.-L. (2022). Data processing of shipborne absolute gravity measurement based on extended Kalman filter algorithm. Acta Phys. Sin..

[32] Huang C.-F., Li A., Qin F.-J., Fang J., Chen X. (2023). An Atomic Gravimeter Dynamic Measurement Method Based on Kalman Filter. Meas. Sci. Technol..

[33] Huang C., Li A., Qin F., Gong W., Che H. (2023). Temperature Drift Modeling and Compensation of Accelerometer Applied in Atom Gravimeter. IEEE Sens. J..

[34] Lautier J., Volodimer L., Hardin T., Merlet S., Lours M., Pereira Dos Santos F., Landragin A. (2014). Hybridizing Matter-Wave and Classical Accelerometers. Appl. Phys. Lett..

[35] Santarelli G., Audoin C., Makdissi A., Laurent P., Dick G.J., Clairon A. (1998). Frequency Stability Degradation of an Oscillator Slaved to a Periodically Interrogated Atomic Resonator. IEEE Trans. Ultrason. Ferroelectr. Freq. Control.

[36] Greenhall C.A. (1998). A Derivation of the Long-Term Degradation of a Pulsed Atomic Frequency Standard from a Control-Loop Model. IEEE Trans. Ultrason. Ferroelectr. Freq. Control.

[37] Lo Presti L., Rovera D., De Marchi A. (1998). A Simple Analysis of the Dick Effect in Terms of Phase Noise Spectral Densities. IEEE Trans. Ultrason. Ferroelectr. Freq. Control.

[38] Audoin C., Santarelli G., Makdissi A., Clairon A. (1998). Properties of an Oscillator Slaved to a Periodically Interrogated Atomic Resonator. IEEE Trans. Ultrason. Ferroelectr. Freq. Control.

[39] Venkataramani R., Bresler Y. (2000). Perfect Reconstruction Formulas and Bounds on Aliasing Error in Sub-Nyquist Nonuniform Sampling of Multiband Signals. IEEE Trans. Inf. Theory.

[40] Joyet A., Mileti G., Dudle G., Thomann P. (2001). Theoretical Study of the Dick Effect in a Continuously Operated Ramsey Resonator. IEEE Trans. Instrum. Meas..

[41] Cheinet P., Canuel B., Pereira Dos Santos F., Gauguet A., Yver-Leduc F., Landragin A. (2008). Measurement of the Sensitivity Function in a Time-Domain Atomic Interferometer. IEEE Trans. Instrum. Meas..

